# Genome Sequence of the Mycorrhiza Helper Bacterium Streptomyces sp. Strain AcH 505

**DOI:** 10.1128/genomeA.01386-14

**Published:** 2015-04-02

**Authors:** M. T. Tarkka, L. Feldhahn, F. Buscot, T. Wubet

**Affiliations:** a Department of Soil Ecology, UFZ-Helmholtz Centre for Environmental Research, Halle, Germany; b German Centre for Integrative Biodiversity Research (iDiv) Halle-Jena-Leipzig, Leipzig, Germany

## Abstract

A draft genome sequence of Streptomyces sp. strain AcH 505 is presented here. The genome encodes 22 secondary metabolite gene clusters and a large arsenal of secreted proteins, and their comparative and functional analyses will help to advance our knowledge of symbiotic interactions and fungal and plant biomass degradation.

## GENOME ANNOUNCEMENT

The filamentous actinobacteria of the genus Streptomyces are ubiquitous in soil (1). They are known as decomposers of organic material and an especially rich source of antimicrobial compounds (2). Members of this genus have evolved to live in symbiosis with plants, fungi, and animals (3, 4). To foster nutrient acquisition, most plants engage in a mutualistic association with filamentous fungi and form mycorrhizas (5). Numerous lineages of bacteria, including the streptomycetes (6), associate with mycorrhizas. Some of these strains are implicated in the stimulation of mycorrhiza formation and are called mycorrhiza helper bacteria (MHB) (7).

Here, we present the draft genome sequence of MHB Streptomyces sp. strain AcH 505, isolated from the vicinity of mycorrhizal roots of Norway spruce in Haigerloch, southwestern Germany (8). Strain AcH 505 stimulates fungal growth by the metabolite auxofuran (9), promotes mycorrhiza formation by enhancing root colonization by fungi, and stimulates plant root branching (10, 11), but also elicits a systemic defense response against powdery mildew (12).

The AcH 505 genome was sequenced at Eurofins (Ebersberg, Germany) using Illumina HiSeq2000 and at the Department of Soil Ecology, UFZ, using 454 Titanium. A total of 25,470,801 single-end Illumina reads with a mean size of 79 bp were assembled by Velvet version 1.2.07 (13) with *k*-mer = {21,29,37,45,53,61} into 222,873 contigs with an average size of 514 bp and an *N*_50_ value of 1,100 bp. These contigs were then assembled with 275,317 paired-end 454 reads, with a mean size of 357 bp, by GS *de novo* assembler version 2.7, generating 27 large scaffolds with an *N*_50_ value of 6,869,175 bp and totaling 9,005,794 bp. The draft genome of AcH 505 shows closest correspondence to the Streptomyces griseus subsp. griseus NBRC 13350 genome. There are 7,822 open reading frames in the AcH 505 genome, among which 5,653 have predicted protein functions and 66 encode RNA. AntiSMASH version 2.0 (14) detected 22 secondary metabolite gene clusters for the synthesis of polyketides, nonribosomal peptides, and terpenes; the gene clusters exhibited extensive genomic synteny to those of other Streptomyces strains. From the fungal and plant cell wall active enzymes, the AcH 505 genome encodes 13 chitinases/chitosanases, eight endoglucanases, a pectate lyase, and three endo-alpha-1-6-d-mannanases. An AcH 505 gene encoding 1-aminocyclopropane-1-carboxylate deaminase and two salicylate esterases may play a role in AcH 505–plant interaction. Functional analyses and comparative studies between AcH 505 and other Streptomyces genomes will now paint a more comprehensive picture into the mechanisms of the mycorrhiza helper effect and plant disease suppression, and help advance our knowledge of symbiotic interactions.

### Nucleotide sequence accession number.

The nucleotide sequence has been deposited at DDBJ/EMBL/GenBank under the accession number JTIY00000000.
